# Alteration in Brain Functional and Effective Connectivity in Subjects With Hypertension

**DOI:** 10.3389/fphys.2018.00669

**Published:** 2018-05-31

**Authors:** Lingguo Bu, Congcong Huo, Gongcheng Xu, Ying Liu, Zengyong Li, Yubo Fan, Jianfeng Li

**Affiliations:** ^1^Key Laboratory of High Efficiency and Clean Mechanical Manufacture, School of Mechanical Engineering, Shandong University, Jinan, China; ^2^Beijing Key Laboratory of Rehabilitation Technical Aids for Old-Age Disability, National Research Center for Rehabilitation Technical Aids, Beijing, China; ^3^Key Laboratory of Rehabilitation Aids Technology and System of the Ministry of Civil Affairs, Beijing, China

**Keywords:** hypertension, functional connectivity, effective connectivity, near-infrared spectroscopy, posture change, wavelet phase coherence, coupling strength

## Abstract

To reveal the physiological mechanism of the cognitive decline in subjects with hypertension, the functional connectivity (FC) was assessed by using the wavelet phase coherence (WPCO), and effective connectivity (EC) was assessed by using the coupling strength (CS) of near-infrared spectroscopy (NIRS) signals. NIRS signals were continuously recorded from the prefrontal cortex, sensorimotor cortex, and occipital lobes of 13 hypertensive patients (hypertension group, 70 ± 6.5 years old) and 16 elderly healthy subjects (control group, 71 ± 5.5 years old) in resting and standing periods. WPCO and CS were calculated in four frequency intervals: I, 0.6–2; II, 0.145–0.6; III, 0.052–0.145; and IV, 0.021–0.052 Hz. CS quantifies coupling amplitude. In comparison with the control group, the hypertension group showed significantly decreased (*p* < 0.05) WPCO and CS in intervals III and IV and in the resting and standing states. WPCO and CS were significantly decreased in the resting state compared with those in the standing state in the hypertension group (*p* < 0.05). Decreased WPCO and CS indicated a reduced network interaction, suggesting disturbed neurovascular coupling in subjects with hypertension. Compared with the control group, the hypertension group showed significantly lower Mini-Mental State Examination (MMSE) (*p* = 0.028) and Montreal Cognitive Assessment (MoCA) scores (*p* = 0.011). In the hypertension group, correlation analysis showed that WPCO and CS were significantly positively correlated with MMSE and MoCA scores, respectively. These findings may provide evidence of impaired cognitive function in hypertension and can enhance the understanding on neurovascular coupling.

## Introduction

Hypertension is a pandemic disease worldwide, particularly affecting 65–75% of individuals older than 65 years. Disruptions in neurovascular coupling are considered to be related to hypertension, which can cause diseases including cognitive decline, stroke, and certain microvascular diseases (Gianaros et al., 2009; Wright and Harding, 2010; Li et al., 2013a, 2014, 2015; Queiroz et al., 2013; Fang et al., 2016; Norris, 2016; Men et al., 2017). Patients with hypertension exhibit poor performance in language, processing speed, visuospatial abilities, attention, executive functioning, and memory (Harrison et al., 2012; Muela et al., 2017; Ticcinelli et al., 2017). The possible underlying mechanisms of hypertension are hypoperfusion and neurodegeneration (Novak and Hajjar, 2010). However, limited evidence has been shown regarding the coupling mechanism of alterations in brain network connectivity in hypertension.

Orthostatic hypertension (OHT) and orthostatic hypotension (OH) are widely observed in elderly people (Wu et al., 2008). Blood pressure, which can be affected by postural change, is related to significant risk of cardiovascular disease (Kario et al., 2002; Tachtsidis et al., 2004). Thus, postural change-related connectivity for the elderly with hypertension is a worthy study (Kario et al., 2002; Gangavati et al., 2011). In fact, posture control is a complex dynamic sensorimotor process rather than a simple summation of static reflexes; any postural behavior requires postural orientation and equilibrium (Horak, 2006). “Postural control” and “brain functions” specifically refer to the functional connectivity (FC) and effective connectivity (EC) during maintenance of standing postures in subjects with hypertension. Analyzing of the brain network connections in subjects with hypertension through different body postures allows an in-depth detection of the functional performance of neurovascular coupling mechanism.

Brain regions commonly work together to form a functional network with a high level of ongoing, strongly correlated spontaneous neurovascular coupling interactions between brain regions in the presence or absence of a task or stimulus (Fox and Raichle, 2007). Historically, owing to the absence of tools for studying the function of distributed networks, traditional localization theories in neurology emphasize the relationship between focal structural damage and behavioral deficits (Carter et al., 2012). Brain activity magnitude for a specific region was significantly higher in subjects with hypertension than those in the healthy subjects (Li et al., 2013b). A study based on near-infrared spectroscopy (NIRS) has demonstrated that the spontaneous oscillations present in the cerebral play a regulatory role in postural control (Tachtsidis et al., 2004). Connectivity-based approaches provide additional insight into network reorganization (Carter et al., 2012). EC, which refers explicitly to the influence that one neural system exerts over another either at a synaptic or population level, can reveal the flow of information between the brain regions (Friston, 2011). FC measures the connection between brain regions and the strength of this connection. One method of characterizing the FC is based on the wavelet phase coherence (WPCO). Numerous previous studies have used this method as basis for evaluating FC between difference regions (Dommer et al., 2012; Bu et al., 2016, 2017). To fully understand the neurovascular coupling interaction among brain regions, we adopted EC, which can reveal the flow of information between the brain regions. EC refers explicitly to the influence that one neural system exerts over another, either at a synaptic or population level (Friston, 2011).

Near-infrared spectroscopy technique can noninvasively and continuously measure brain activation by monitoring changes in local oxygenated (Δ[HbO_2_]) and deoxygenated (Δ[HHb]) hemoglobin concentrations (Tak et al., 2010; Cui et al., 2011; Sasai et al., 2011; Scholkmann et al., 2014; Iso et al., 2016). This technology provides certain advantages, such as portability, convenience, cost effectiveness, and low constraints on test subjects, in research on subjects and in the study of brain network (Carter et al., 2010; Kirilina et al., 2012; Yamasaki et al., 2013; Bajaj et al., 2014; Sørensen et al., 2014; Yeung et al., 2016). NIRS has been applied in studies on subjects with hypertension. Compared with that in healthy elderly, WPCO is significantly lower between left and right prefrontal regions in elderly subjects with hypertension in resting state (Li et al., 2014). The amplitudes of Δ[HbO_2_] are significantly higher in hypertensive patients than those in the healthy subjects (Li et al., 2013b).

Functional resting-state networks are widely distributed in the brain (Sorg et al., 2007; Li et al., 2017). Several brain regions must work together to process and integrate information and develop the brain’s advanced features (Leech et al., 2011). Advanced neural information processing functions, such as judgment and analysis, are performed in the prefrontal cortex (Miller and Cohen, 2001). The motor cortex plays an important role in recovery from motor dysfunction (Nudo, 2006). The occipital lobe, which contains most of the anatomical regions of the visual cortex, is the visual processing center of the brain (Salvador et al., 2005). Standing activated the prefrontal cortex, the cerebellar anterior lobe, and the right visual cortex (Ouchi et al., 1999). However, the reorganization of interregional interactions on the brain network in subjects with hypertension under different posture states remains poorly understood.

We hypothesized that brain network connections may be disturbed in response to posture-related changes (resting to standing) in subjects with hypertension. In this paper, FC was assessed by using the WPCO and EC was assessed by using the coupling strength (CS) based on a coupled-phase-oscillator model and dynamic Bayesian inference. Our study further addressed the following points: (1) alterations in FC and EC among different brain regions in hypertension and control groups under different postural states; and (2) correlation analysis was used to reveal the relationship between brain network connections and cognitive performance, which assessed Mini-Mental State Examination (MMSE) and Montreal Cognitive Assessment (MoCA) scale.

## Materials and Methods

### Subjects

A total of 29 subjects, including 13 hypertensive patients (age: 70 ± 6.5 years; hypertension group) and 16 elderly healthy subjects (age: 71 ± 5.5 years; control group), were recruited from a local community. All participants satisfied the following criteria: (1) no structural abnormalities; (2) no neurological or psychiatric disorders; (3) no use of medication; and (4) educational level of senior high school or above. In this study, a diagnosis of hypertension was performed when systolic blood pressure ≥140 mm Hg or diastolic blood pressure ≥90 mm Hg (Jones et al., 2003). The experimental procedure was approved by the Human Ethics Committee of National Research Center for Rehabilitation Technical Aids and was in accordance with the ethical standards specified by the Helsinki Declaration of 1975 (revised in 2008). Basic information on our subjects, including age, systolic, and diastolic blood pressures, and MMSE and MoCA scores were recorded before the test (**Table 1**). All participants provided written informed consent before participating in the study.

**Table 1:** Basic information on the NIRS experimental subjects.

Condition	Hypertension	Control
	group	group
Age (years)	70 ± 6.5	71 ± 5.5
Systolic blood pressure (mm Hg)	153 ± 11.9**	120.1 ± 8.8
Diastolic blood pressure (mm Hg)	97.1 ± 7.3**	72.2 ± 5.6

*Values are presented as means and SDs and percentages. *p*-Values for differences are calculated using *t*-test. ^∗∗^*p <* 0.01.*

### Cognitive Assessment Through Questionnaire

Mini-Mental State Examination and MoCA were applied to assess cognitive function between hypertension and control groups.

#### MMSE

Mini-Mental State Examination is a commonly used 30-point scale to assess cognitive function in orientation, attention, calculation, recall, language, and praxis (Muela et al., 2017). This scale is also utilized for cognitive assessment in an office space or at bedside (Harrell et al., 2000).

#### MoCA

Montreal Cognitive Assessment is an assessment tool for attention and concentration; this tool offers high sensitivity for the rapid screening of mild cognitive impairment (Nasreddine et al., 2005). The highest total possible score is 30 points.

### Experimental Measurements

Given that diurnal patterns can significantly alter blood pressure, all examinations were performed in the morning (starting at 8 a.m. and finishing at 11:30 a.m. at the latest). All NIRS signal acquisition procedures were conducted within 5 days. In this study, laboratory temperature was controlled at 22°C. Before NIRS signal acquisition, all samples were evaluated by MMSE and MoCA. The NIRS test was divided into two parts, namely, resting and standing periods. During the first part of the test, each subject took a 10-min resting period. During this period, the participants were required to close their eyes and relax, and moving was avoided (Gusnard and Raichle, 2001). After the 10-min resting period, the 10-min standing period started within 10 s.

**FIGURE 1:**
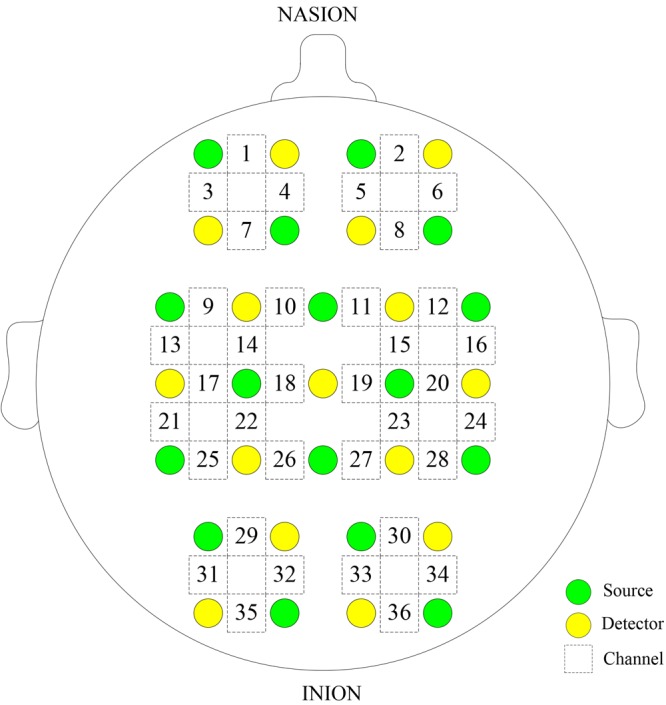
Configuration of the measurement channels in the prefrontal cortex, sensorimotor cortical, and occipital lobe areas based on the international 10/10 system.

Δ[HbO_2_] and Δ[HHb] of NIRS signals were acquired using a multi-channel commercial NIRS system (NirSmart, Danyang Huichuang Medical Equipment Co., Ltd., China) at the sampling rate of 10 Hz. The wavelengths used were 780, 808, and 850 nm, and 36 channels were contributed by sensors and optodes (**Figure 1**). NIRS measures cortical activities from the head surface without anatomical information of the brain. NIRS signals were recorded from 36 channels consisting of 16 light sources and 15 detectors and equally distributed over both hemispheres. The calibration function of the instrument and the corresponding template were used to ensure that the channels fall exactly in correspondence with the 10/10 electrode positions with the different head size of the participants (Koessler et al., 2009). The 36 channels were positioned over the left prefrontal cortex (LPFC), right prefrontal cortex (RPFC), left motor cortex (LMC), right motor cortex (RMC), left occipital lobe (LOL), and right occipital lobe (ROL).

### Data Preprocessing

For improving the accuracy of experimental results, NIRS signals denoizing was conducted by using the method described in our previous studies (Li et al., 2013b; Bu et al., 2017). A method based on moving standard deviation and spline interpolation was used to reduce movement artifacts in this study (Scholkmann et al., 2010). By setting a certain window length W, the moving standard deviation was calculated. For identifying the segmented Xma containing movement artifacts, the moving standard deviation time series *s*(*t*) was processed by using a threshold value *T*. Xma was used to subtract the cubic spline difference function so as to achieve the effect of artifact. In all cases, parameter *p* was set to *p* = 0.01. This method can be adapted to different types of movement artifacts. A Butterworth filter was selected to filter noise and interference signals (Bu et al., 2017).

### Wavelet Transform

Wavelet transform is a method that transforms signals from the time domain to the time–frequency domain by using frequency-adjustable filtering window; the output is a three-dimensional map containing time–frequency–amplitude information. WT method has been described previously in detail (Han et al., 2014).

The instantaneous relative phase for each frequency and time can be obtained based on WT. Phase dynamic information can be used to investigate the coupling relationships among the oscillations from different signals in difference time scales (Bernjak et al., 2012).

These oscillation signals measured by NIRS implied different physiological meanings at different frequency intervals, reflecting myogenic, neurogenic, or metabolic regulation of microvascular blood flow (Han et al., 2014; Vermeij et al., 2014). Four major frequency-specific oscillator intervals are distinguished by WT in the intervals as follows: I: 0.6–2 (cardiac activity); II: 0.145–0.6 (respiratory activity); III: 0.052–0.145 (myogenic activity); and IV: 0.021–0.052 (neurogenic activity) (Shiogai et al., 2010).

### Wavelet-Based Coherence Analysis

This method was described in our previous studies (Bu et al., 2016; Wang B. et al., 2016; Wang W. et al., 2016). WPCO is used to determine phase coherence by calculating the phase difference of signal after the wavelet transform (Veber et al., 2004; Sheppard et al., 2011). The result explains the change characteristics of the connection between different brain regions in terms of phase congruency (Bandrivskyy et al., 2004; Bernjak et al., 2012). In this study, for each subject, WPCO values among all possible pairs of 36 channels were calculated for the specific posture state and frequency intervals.

Wavelet phase coherence value ranges from 0 to 1. The value of 0 indicates that the fluctuation of the two time series is completely out of synchronization; otherwise, the value of 1 indicates complete synchronization. Amplitude-adjusted Fourier transform (AAFT) was adopted in this study to generate 100 surrogate signals, which can be used to test the significance of WPCO (Wang B. et al., 2016).

### Effective Connectivity

The master-slave coupling between the blood oxygen signal Δ[HbO_2_] oscillations in different brain regions exhibits various degrees. EC reveals CS between the brain regions based on the phase dynamic (Friston, 2011; Ticcinelli et al., 2017).

A network of *N*-coupled oscillator system can be decomposed as *N* stochastic differential equations with time parameter *t* (Schwabedal and Pikovsky, 2010; Ticcinelli et al., 2017):

(1) $$\begin{array}{c} \overset{\cdot }{\phi_{i}}(t)\;=\;\omega_{i}(t)\;+\;q_{i}(\phi_{i},\phi_{j},\phi_{k},\ldots ,\phi_{N},t)+\xi_{i}(t), \end{array}$$

where *i* = 1, 2, …, *N*, and the natural frequency of each oscillator is defined as ω_i_(t). The coupling function, which is influenced by phases ϕ_1,…,N_, is defined as *q_i_* and can be formed by the sum of different coupling orders (Stankovski et al., 2016). ξ_i_(t) represents Gaussian white noise. Magnitude changes of *q_i_* were added to ω_i_ and thus contributed to the overall frequency changes in the second oscillator. With Fourier approximation, Equation (1) for each oscillator is decomposed into a sum of base functions (Stankovski et al., 2015), which is modulated by time-varying parameter $c_{k}^{\left(i\right)}$ (Stankovski et al., 2015):

(2) $$\begin{array}{c} \overset{\cdot }{\phi_{i}}(t)=\sum_{k=-K}^{K}c_{k}^{\left(i\right)}\Phi_{k}(\phi_{1},\phi_{2},\ldots ,\phi_{N})+\zeta_{i}(t), \end{array}$$

where Φ_k_ = exp[1(k_1_ϕ_1_ + k_2_ϕ_2_ + … + k_N_ϕ_N_)] (Stankovski et al., 2015). $c_{k}^{\left(i\right)}$ provides information on the coupling function. $c_{k}^{\left(i\right)}$ can be inferred by dynamical Bayesian inference, which calculates the posterior parameter recursively by using the likelihood function and the given information (Stankovski et al., 2015). For each subject, coupling interactions among all possible pairs of 36 channels were computed for the specific posture state and frequency intervals. A total of 100 AAFT surrogate signals were used to calculate the mean surrogate CS value for evaluating the significance of the CS value in each frequency interval.

### Statistical Analysis

Data on the results were tested for normality (Kolmogorov–Smirnov test) and homogeneity of variance (Levene test) to ensure that they meet the assumption for parameter analysis. To test for the significance of the effect of interest (the main effect of both group and condition as well as the interaction of group × condition), a two-way repeated measures ANOVA was carried out on each brain network connections; group (hypertension and controls) as a between-subject factor and condition (resting and standing) as a repeated measure factor.

One-way ANOVA was conducted to compare the differences in WPCO values and CS between hypertension and control groups in the resting and standing periods, respectively. Repeated measures analysis was used to compare the differences in WPCO values and CS between resting and standing periods in hypertension and control groups. An independent Student’s *t*-test has been conducted in MMSE and MoCA scores between hypertension and control groups. To identify the association between the brain network connections and the cognitive performance of subjects with hypertension, we extracted and correlated the interregional connection parameters with the MMSE and MoCA scores using Pearson’s correlative analysis. *p <* 0.05 was considered statistically significant.

## Results

### Functional Connectivity

In interval III, the WPCO of LPFC–RPFC showed significant main effect of condition (*F =* 4.944, *p =* 0.035) and interaction between two factors (*F =* 5.34, *p =* 0.029); the WPCO of LMC–RMC showed significant main effect of group (*F =* 9.04, *p =* 0.006) and effect of condition (*F =* 7.143, *p =* 0.013). The WPCO values in interval IV showed significant main effect of condition in connections of LPFC–LMC (*F =* 4.704, *p =* 0.039) and LMC–LOL (*F =* 4.726, *p =* 0.048).

#### Hypertension-Related Change in WPCO

**FIGURE 2:**
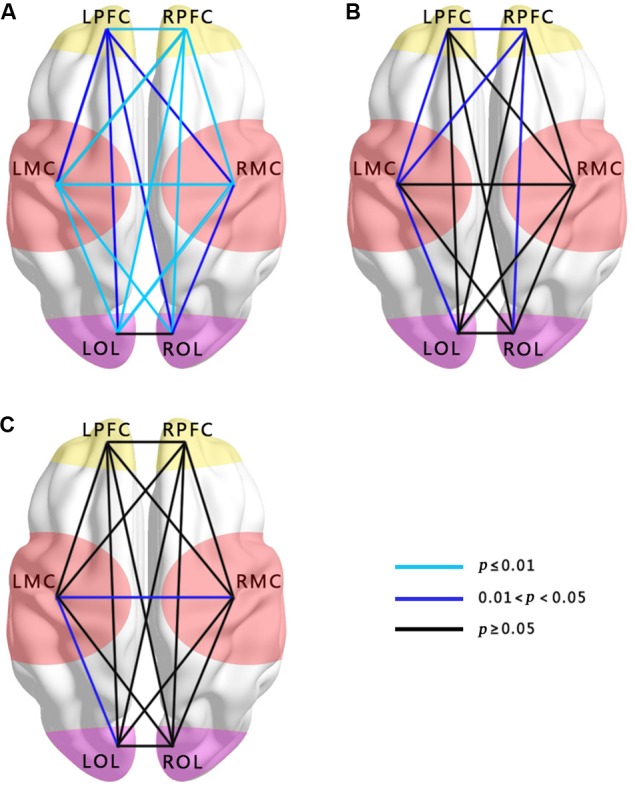
Comparisons of WPCO in the 15 connectivity types among the groups. **(A)** Comparison between hypertension and control groups at rest in interval III. **(B)** Comparison between hypertension and control groups at rest in interval IV. **(C)** Comparison between hypertension and control groups at stand in interval III. Black lines represent no significant differences of WPCO values (*p* > 0.05). Dark blue represents the significance of the WPCO value (0.01 < *p* < 0.05). Light blue lines represent strong significance of the WPCO value (*p* ≤ 0.01).

In **Figures 2A,B**, the WPCO value of LPFC–RPFC (*F =* 9.32, *p <* 0.01), LPFC–LMC (*F =* 5.741, *p =* 0.024), LMC–RMC (*F =* 9.031, *p <* 0.01), LPFC–RMC (*F =* 4.292, *p =* 0.048), LPFC–LOL (*F =* 5.271, *p =* 0.03), LPFC–ROL (*F =* 5.056, *p =* 0.033), RPFC–LMC (*F =* 12.771, *p <* 0.01), RPFC–RMC (*F =* 10.697, *p <* 0.01), RPFC–LOL (*F =* 17.168, *p <* 0.01), LMC–ROL (*F =* 10.671, *p <* 0.01), RPFC–ROL (*F =* 15.737, *p <* 0.01), LMC–LOL (*F =* 15.303, *p <* 0.01), RMC–LOL (*F =* 10.274, *p <* 0.01), and RMC–ROL (*F =* 6.738, *p =* 0.015) in interval III and LPFC–LMC (*F =* 4.412, *p =* 0.045), RPFC–LMC (*F =* 5.251, *p =* 0.03), RPFC–ROL (*F =* 6.129, *p =* 0.02), and LMC–LOL (*F =* 5.049, *p =* 0.033) in interval IV were significantly lower in the hypertension group than those in the control group in the resting period.

In the standing period, the WPCO values of LMC–RMC (*F =* 5.443, *p =* 0.027) and LMC–LOL (*F =* 5.534, *p =* 0.026) in interval III were also significantly lower in the hypertension group than those in the control group (**Figure 2C**).

#### Posture-Related Change in WPCO

Through repeated measures analysis, the WPCO values of LPFC–RPFC (*F* = 5.134, *p =* 0.003), LMC–RMC (*F* = 4.424, *p =* 0.007), LPFC–LMC (*F* = 5.032, *p =* 0.004), LPFC–RMC (*F* = 3.727, *p =* 0.017), LPFC–LOL (*F* = 3.488, *p =* 0.022), LPFC–ROL (*F* = 3.488, *p =* 0.022), RPFC–LMC (*F* = 5.863, *p =* 0.002), RPFC–RMC (*F* = 5.034, *p =* 0.004), RPFC–LOL (*F* = 5.424, *p =* 0.002), RPFC–ROL (*F* = 5.827, *p =* 0.002), LMC–LOL (*F* = 5.945, *p =* 0.001), LMC–ROL (*F* = 3.835, *p =* 0.015), RMC–LOL (*F* = 4.193, *p =* 0.01), and RMC–ROL (*F* = 2.963, *p =* 0.04) show significant lower from resting period to standing period in interval III.

#### Connectivity Maps

**FIGURE 3:**
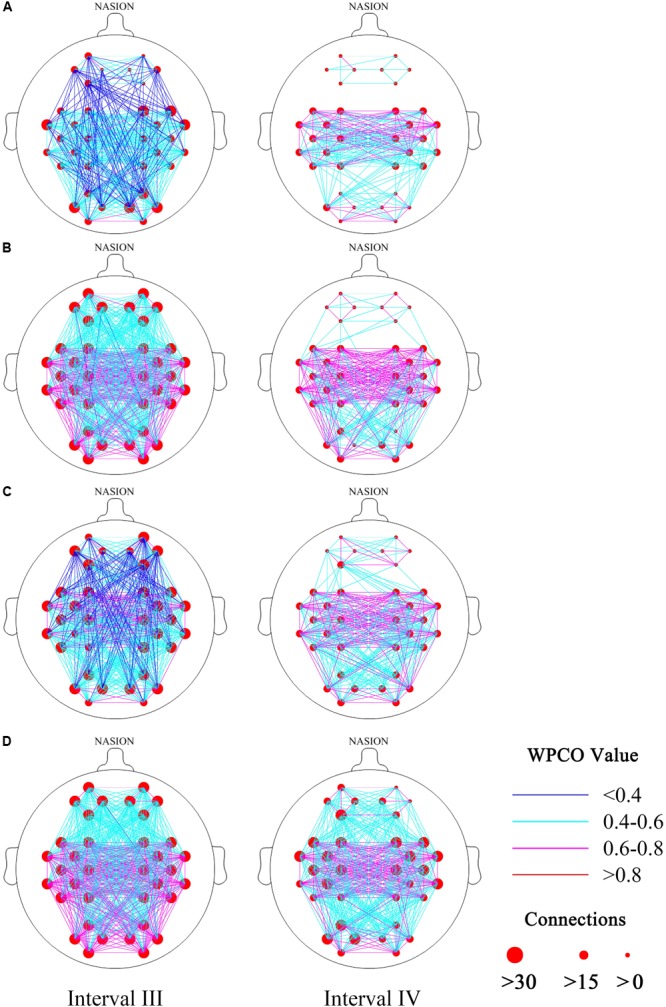
Functional connectivity maps (frequency intervals III and IV) revealed by wavelet phase coherence. **(A)** Hypertension group at rest; **(B)** control group at rest; **(C)** hypertension group at stand; and **(D)** control group at stand. Two frequency intervals: III (0.052–0.145 Hz) and IV (0.021–0.052 Hz).

**Figures 3A–D** show the FC of the hypertension and control groups in the two frequency bands during the resting and standing states. The connections in intervals III and IV were less in the hypertension group than in the control group in the resting and standing states. Connections were noted among LPFC–LMC, LPFC–RMC, RPFC–LMC, and RPFC–RMC in the control group at rest, whereas no connection was found in these four types of connection in the hypertension group at rest.

### Effective Connectivity

The CS levels in interval III showed significant main effect of group in connections of LPFC to LOL (*F =* 4.622, *p =* 0.041), LMC to RPFC (*F =* 4.637, *p =* 0.04), RPFC to LOL (*F =* 7.22, *p =* 0.012), RPFC to ROL (*F =* 5.418, *p =* 0.028), and ROL to RPFC (*F =* 7.778, *p =* 0.01). The CS levels in interval IV showed significant main effect of condition in connections of LPFC to RPFC (*F =* 15.683, *p* < 0.001), RPFC to LPFC (*F =* 5.475, *p =* 0.027), LMC to RMC (*F =* 5.263, *p =* 0.03), LPFC to LMC (*F =* 6.023, *p =* 0.021), LPFC to RMC (*F =* 7.17, *p =* 0.012), RPFC to LMC (*F =* 4.455, *p =* 0.044), RMC to LOL (*F =* 11.808, *p =* 0.002), and LOL to RMC (*F =* 6.279, *p =* 0.019).

**FIGURE 4:**
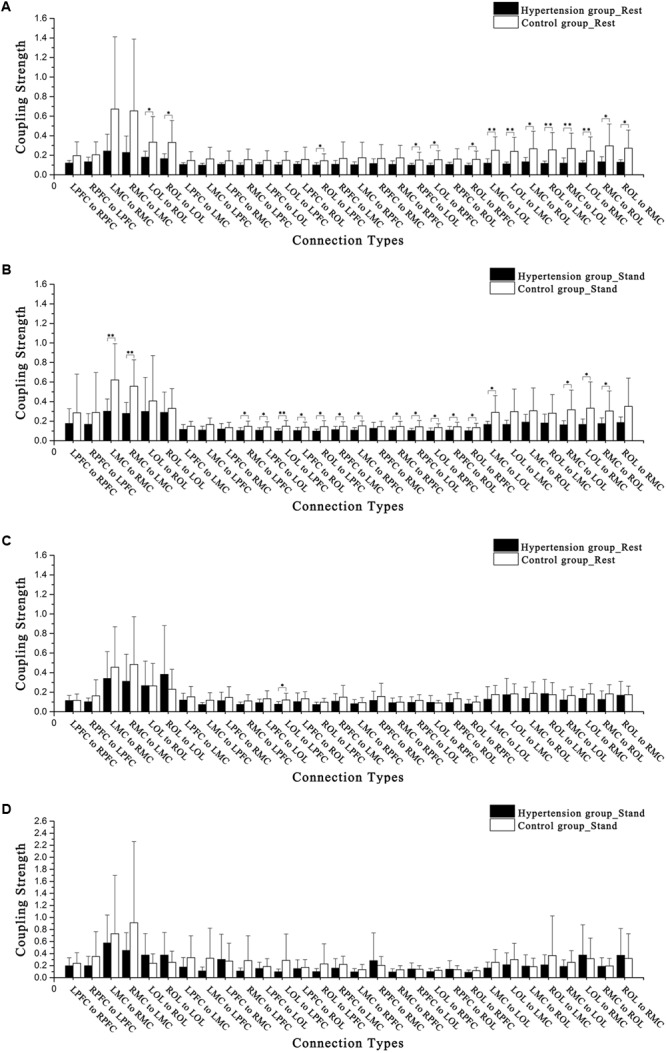
Comparisons of coupling strength in intervals III and IV in the 15 connectivity types. **(A)** Hypertension and control groups at rest in interval III. **(B)** Hypertension and control groups while standing in interval III. **(C)** Hypertension and control groups at rest in interval IV. **(D)** Hypertension and control groups while standing in interval IV. Significant differences are marked with ^∗^*p <* 0.05 or ^∗∗^*p <* 0.01.

**Figures 4A–D** show the CS in intervals III and IV in the hypertension and control groups in the resting and standing states. In the resting period, the CS levels of LOL to ROL (*F =* 4.257, *p =* 0.049), ROL to LOL (*F =* 6.822, *p =* 0.015), ROL to LPFC (*F =* 4.432, *p =* 0.045), RPFC to LOL (*F =* 5.402, *p =* 0.028), LOL to RPFC (*F =* 4.913, *p =* 0.035), ROL to RPFC (*F =* 6.211, *p =* 0.019), LMC to LOL (*F =* 11.483, *p <* 0.01), LOL to LMC (*F =* 10.046, *p <* 0.01), LMC to ROL (*F =* 7.009, *p =* 0.013), ROL to LMC (*F =* 7.97, *p <* 0.01), RMC to LOL (*F =* 10.762, *p <* 0.01), LOL to RMC (*F =* 8.873, *p <* 0.01), RMC to ROL (*F =* 6.594, *p =* 0.016), ROL to RMC in interval III (*F =* 7.64, *p =* 0.01), and LOL to LPFC (*F =* 4.563, *p =* 0.042) in interval IV were significantly lower in the hypertension group than those in the control group.

In the standing period, the CS levels of LMC to RMC (*F =* 8.855, *p* < 0.01), RMC to LMC (*F =* 11.921, *p* < 0.01), RMC to LPFC (*F =* 6.067, *p =* 0.02), LPFC to LOL (*F =* 4.572, *p =* 0.042), LOL to LPFC (*F =* 7.965, *p* < 0.01), LPFC to ROL (*F =* 4.454, *p =* 0.044), ROL to LPFC (*F =* 6.701, *p =* 0.015), RPFC to LMC (*F =* 4.711, *p =* 0.039), LMC to RPFC (*F =* 7.539, *p =* 0.011), RMC to RPFC (*F =* 4.993, *p =* 0.034), RPFC to LOL (*F =* 4.402, *p =* 0.045), LOL to RPFC (*F =* 7.174, *p =* 0.012), RPFC to ROL (*F =* 4.67, *p =* 0.04), ROL to RPFC (*F =* 4.793, *p =* 0.037), LMC to LOL (*F =* 6.533, *p =* 0.017), RMC to LOL (*F =* 7.313, *p =* 0.012), LOL to RMC (*F =* 4.793, *p =* 0.037), and RMC to ROL (*F =* 4.921, *p =* 0.035) in interval III were significantly lower in the hypertension group than those in the control group.

Through repeated measures analysis, the CS of LPFC to LMC denotes the interaction between groups (hypertension and control) and states (resting period and standing period). In interval III, between subjects in the hypertension and control groups and from resting to standing periods, significant differences in connections were observed from LPFC to LMC (*F* = 2.971, *p =* 0.04), LMC to RPFC (*F* = 4.034, *p =* 0.012), RMC to RPFC (*F* = 4.003, *p =* 0.012), LOL to RPFC (*F* = 4.665, *p =* 0.006), ROL to RPFC (*F* = 5.403, *p =* 0.003), LMC to LOL (*F* = 4.447, *p =* 0.007), LMC to ROL (*F* = 3.151, *p =* 0.032), RMC to LOL (*F* = 4.912, *p =* 0.004), and RMC to ROL (*F* = 4.879, *p* = 0.004).

### Correlation Analysis Among Interregional FC, EC, and Cognitive Performance

MMSE (*p =* 0.028) and MoCA scores (*p =* 0.011) were significantly lower in the hypertension group than those in the control group. In the hypertension group, strong positive correlation was observed between MMSE scores and WPCO of LPFC–RPFC in intervals III (*r* = 0.597, *p* = 0.031) and IV (*r* = 0.688, *p* = 0.009). Moreover, MoCA scores and WPCO of LPFC–RPFC in frequency intervals III (*r* = 0.659, *p* = 0.014) and IV (*r* = 0.71, *p* = 0.007) show strong positive correlation. In interval III, strong positive correlations were observed between MMSE scores and the CS from LPFC to RPFC (*r* = 0.684, *p* = 0.01), from RPFC to LPFC (*r* = 0.72, *p* = 0.005), from LPFC to RPFC (*r* = 0.743, *p* = 0.004), and from RPFC to LPFC (*r* = 0.796, *p* = 0.001). Correlation analysis also revealed that the MoCA scores were significantly positive correlated with the CS from RPFC to LPFC in interval III (*r* = 0.585, *p* = 0.036), from LPFC to RPFC (*r* = 0.818, *p* = 0.001), and from RPFC to LPFC (*r* = 0.805, *p* = 0.001) in interval IV.

## Discussion

The findings of this study are as follows: (1) FC and EC among regions in intervals III and IV were significantly lower in hypertension group than that in control group both in resting and standing states. (2) Significantly positive correlation has been found between cognitive scores and connectivity parameters.

Based on neural–vascular coupling, NIRS is a kind of blood oxygen level-dependent (BOLD) technology that detects the blood oxygen parameter Δ[HbO_2_] in brain tissue to indirectly reflect the activity of brain tissue. The activity of the brain nerve is highly correlated with the degree of changes in the blood oxygen content of the brain. Given this correlation, we can infer the activity of brain nerve by measuring the changes of local cerebral blood oxygen and then analyze the synergy between various human brain regions during task implementation. The neurovascular unit is the basic functional unit responsible for neurovascular coupling (Scholkmann et al., 2014).

### Functional Connectivity

In the resting period, except LOL–ROL, the WPCOs in all of the 14 other connection types in interval III were significantly lower in the hypertension group than those in the control group. A functional magnetic resonance imaging (fMRI) study also showed that the patterns of brain in the hypertensive group changed compared with the control group (Li et al., 2015). Our results are consistent with this finding. As described in our previous studies, cerebral oscillation in interval III originated from the intrinsic myogenic activity of smooth muscle cells in resistance vessels (Rowley et al., 2007; Shiogai et al., 2010). The reduction in WPCO values in interval III indicates that hypertension leads to a reduction of phase synchronization in these brain regions. This impaired connectivity may be the vascular or metabolic dysfunction in this region that is attributed to hypertension. The local cerebral blood flow in certain brain regions, including the anterior cingulated cortex and the left posterior cingulate cortex, possibly decreases in certain elderly patients with hypertension (Son et al., 2015). Hypertension also causes vascular stiffening (Iadecola and Davisson, 2008). All of these factors can provide insight into the decrease in phase synchronization caused by hypertension. Hypertension can induce the decline in cognitive function and may cause severe vascular diseases due to the impairment of vascular reserve and microvascular disease.

In the resting period, the WPCO values of LPFC–LMC, RPFC–LMC, RPFC–ROL, and LMC–LOL in interval IV were significantly lower in the hypertension group compared with those in the control group. The IV frequency segment reflects the role of neurogenic activity, which is determined by the combined action of the neurovascular coupling and sympathetic nerve regulation. As demonstrated in precious studies, interval IV is regulated by the neural control of smooth muscle cells within the brain (Zhang et al., 2002; Shiogai et al., 2010). The hypertension group exhibits the most significant loss of coherent connection in frequency IV. Therefore, hypertension primarily damages the brain network by affecting the capability of nerve activities to regulate blood oxygen concentration in the local brain tissue. Hypertension changes the structure of the cerebral blood vessel, which facilitates vascular occlusions and compromises cerebral perfusion (Girouard and Iadecola, 2006). Several factors are conducive to hypertrophy in cerebral arteries and arterioles. The development of cerebrovascular hypertrophy requires sympathetic perivascular innervation, which exerts a trophic effect on the vascular wall (Iadecola and Davisson, 2008). Motion-activated vasomotor center reduces the sympathetic nerve excitability and then decreases blood pressure by the effects of the brain cortex and diencephalon and other advanced nerve centers. Our previous study found that different postures can significantly affect WPCO values in healthy older adults (Wang B. et al., 2016), and our present results are consistent with such finding.

### Effective Connectivity

In the resting period, the CS levels of LOL to ROL, ROL to LOL, ROL to LPFC, RPFC to LOL, LOL to RPFC, ROL to RPFC, LMC to LOL, LOL to LMC, LMC to ROL, ROL to LMC, RMC to LOL, LOL to RMC, RMC to ROL, and ROL to RMC in interval III were significantly lower in the hypertension group than those in the control group. CS is regulated by the myogenic activity in this frequency interval; myogenic activity is produced by the metabolic mechanism of smooth muscle cells, which guarantee the normal contraction and expansion of blood vessels (Shiogai et al., 2010). In this paper, EC indicates the effect of the cerebral cortex neurovascular coupling interactions between brain regions, which is caused by myogenic activity, in one brain region on the cerebral cortex activity in another brain region (Friston, 2011). Whether in the resting state or standing state, the CS of hypertensive patients in prefrontal cortex, sensorimotor cortex, and occipital lobe decreased, which may be due to the changes in mediation mechanism of myogenic activity caused by hypertension. In the hypertension group, the CS levels of ROL to LOL, LMC to LOL, LOL to LMC, LMC to ROL, ROL to LMC, RMC to LOL, LOL to RMC, and ROL to RMC in interval III were significantly lower in the resting period compared with those in the standing period. The decline in CS indicates reduced efficiency of information transmission. Various indices of vascular and brain health are associated with cognitive deficits in hypertensive patients.

The CS of LOL to LPFC in interval IV was significantly lower in the hypertension group than that in the control group in the resting period. The CS levels of RPFC to LPFC and RMC to LPFC in interval IV were significantly lower in resting period than those in standing period in the hypertension group. In the control group, the CS levels of LPFC to RPFC and LOL to RPFC in interval IV were significantly lower in resting period than those in standing period. Frequency interval IV may reflect the role of neurogenic activity (Shiogai et al., 2010). Postural orientation and equilibrium are needed in any postural behavior (Horak, 2006). Sensation and balance are required in a sit-to-stand posture change in elderly people (Lord et al., 2002). Hypertension primarily damages the brain network by affecting the capability of neurogenic activity to mediate oxygen concentration in local brain tissue. Neural activity is achieved through the coupling function of the neurovascular and the automatic adjustment of the sympathetic nerves. The neurogenic mechanism of the sympathetic nervous system plays a key role in the occurrence and development of hypertension. A study shows that the mechanism may be related to visual phenomena, including visual loss and hallucinations, to suggest occipital lobe origin (Stott et al., 2005). Hypertension affects disruptions in neurovascular coupling in the brain regions, including the prefrontal cortex, sensorimotor cortex, and occipital lobe (Novak and Hajjar, 2010). Combined with the above explanations, CS in interval IV was significantly lower in LOL to LPFC at rest and RPFC to LPFC and RMC to LPFC while standing in the hypertension group. As a result of this complex posture change, CS in interval IV was significantly lower in resting state than that in the standing state in RPFC to LPFC and RMC to LPFC of the hypertension group and LPFC to RPFC and LOL to RPFC of the control group. Our previous study found that the activity level was higher in the hypertension group than those in the control group (Li et al., 2013b). High cerebral oscillations are verified to be associated with the development of stroke risk (Li et al., 2013b). High cerebral oscillations in interval IV indicate a neurogenic response to systemic high blood pressure.

Compared with those in the control group, the scores of MMSE and MoCA were lower in the hypertension group. Correlation analysis showed that connectivity parameters were significantly positively related with cognitive performance. Blood pressure is inversely proportional to MMSE and MoCA scores (Muela et al., 2017). The modulation of neurovascular coupling can guarantee rapid spatial and temporal increases in cerebral blood flow in response to neuronal activation (Kasischke et al., 2004). The disruption of neurovascular coupling in hypertension leads to homeostatic imbalance, which may contribute to brain dysfunction. These results may indicate that decreased connectivity among the brain regions may be an evidence of impaired cognitive function in the hypertension group.

### Limitations

Near-infrared spectroscopy is known to present a systemic artifact. Certain groups implement a short channel or spatial regression to separate systemic effect from cortical signals (Gagnon et al., 2012; Kirilina et al., 2012). However, none of the above methods was used in this study, thereby constituting a study limitation.

Given that physiological noise is induced in NIRS measurement, Mayer waves may affect FC and EC. The interference of Mayer waves should be taken into account in a future study. In addition, the number of subjects included in the present work was relatively limited in light of the current standards for neuroimaging experiments (Friston, 2012).

## Conclusion

Wavelet phase coherence was used to assess the phase synchronization of Δ[HbO_2_] between LPFC, RPFC, LMC, RMC, LOL, and ROL during resting and standing periods in subjects with hypertension. Coupling function based on dynamic Bayesian inference was applied to evaluate the changes in CS. The WPCO and CS in intervals III and IV were significantly lower in the hypertension group than those in the control group. These values were also significantly lower in the resting state than those in the standing state in the hypertension group. The decreased WPCO and CS indicated disturbed coupling relationship among the brain regions in the subjects with hypertension. Correlation analysis also revealed significantly positive correlation between cognitive scores and connectivity parameters. Correlation analysis also revealed significantly positive correlation between cognitive scores and connectivity parameters. Decreased connectivity may be indicative of impaired cognitive function. These results provide an evidence for cognitive decline and can aid in detecting the functional performance of neurovascular coupling mechanism in subjects with hypertension.

## Author Contributions

ZL and YF designed the study and edited the manuscript. LB did the experiment, analyzed the data, contributed to the physiological interpretation of the results, and drafted the manuscript. CH and GX did the experiment and analyzed the data. YL performed the statistical analysis. JL administrated this project.

## Conflict of Interest Statement

The authors declare that the research was conducted in the absence of any commercial or financial relationships that could be construed as a potential conflict of interest.
